# Awareness and Attitude Towards MRONJ Among Physicians Prescribing Antiresorptive Drugs: A Cross-Sectional Study

**DOI:** 10.1186/s12903-025-06490-5

**Published:** 2025-07-02

**Authors:** Murat Mutlu, Serpil Altundoğan, Ömer Faruk Kocamaz

**Affiliations:** Faculty of Dentistry, Department of Oral and Maxillofacial Surgery, Ankara University, Ankara, Türkiye

**Keywords:** Bisphosphonate, Denosumab, Medication-related osteonecrosis of the jaw, Awareness

## Abstract

**Background:**

MRONJ (Medication-Related Osteonecrosis of the Jaw) refers to a condition characterized by osteonecrosis of the jawbone associated with the use of certain medications. One of the most common causes of MRONJ is bisphosphonates, which are frequently used today to treat osteoporosis, metabolic bone diseases, and the skeletal effects of malignancies.

**Objective:**

The aim of this study is to assess the awareness and approaches of medical doctors who prescribe bisphosphonate group drugs regarding MRONJ.

**Method:**

A total of 125 physicians specializing in the departments of oncology, orthopedics and traumatology, physical medicine and rehabilitation, and obstetrics and gynecology participated in our study. The participants completed questionnaire forms consisting of 15 questions related to MRONJ, and the results were analyzed.

**Results:**

The specialists who participated in the study were found to prescribe zoledronate and alendronate more frequently than other antiresorptive medications. Over the past year, when examining the frequency of encountering patients with MRONJ, 56% of physicians had not encountered any cases, while 44% had encountered between one and four cases. The group that most frequently requested consultations from physicians regarding bisphosphonates was identified as dentists, with a rate of 50.4%, followed by oral and maxillofacial surgeons at 36.8%. The specialists who participated in the study reported that when they encounter patients with osteonecrosis, 39.2% most frequently referred them to oral and maxillofacial surgery.

**Conclusion:**

To enhance awareness of MRONJ, a multidisciplinary approach should be adopted between medical and dental disciplines, and a well-coordinated consultation system should be established. This will ensure that patients undergo dental examinations and complete necessary interventional dental procedures before commencing antiresorptive drug therapy. Furthermore, implementing educational strategies such as academic publications, training seminars, and symposia is of great importance to increase the level of awareness on this issue.

**Supplementary Information:**

The online version contains supplementary material available at 10.1186/s12903-025-06490-5.

## Introduction

Medication-related osteonecrosis of the jaw (MRONJ) is a condition characterized by exposed bone in the maxillofacial region that persists for more than eight weeks in patients who have not received radiation therapy to the head and neck area but have undergone or are undergoing treatment with antiresorptive or antiangiogenic medications [1]. In an experimental study (2003) involving 36 cases, Marx and Stern examined areas of exposed bone that appeared in the maxilla and mandible of patients undergoing bisphosphonate therapy. Following this research, the concept of bisphosphonate-related osteonecrosis of the jaw (BRONJ) was first defined by Marx and Stern in 2007 [2]. Both denosumab and bisphosphonates are commonly used in patients with osteoporosis to minimize the incidence of bone fractures. The American Association of Oral and Maxillofacial Surgeons (AAOMS) has reported that cases of osteonecrosis in the maxilla and mandible may be associated not only with bisphosphonates but also with antiresorptive and antiangiogenic drugs. In an article published in 2014, the previously used term BRONJ (bisphosphonate-related osteonecrosis of the jaw) was replaced with a more inclusive definition, MRONJ (medication-related osteonecrosis of the jaw)[1].

Although MRONJ is a relatively rare complication of antiresorptive and antiangiogenic medications, it is projected that the older population will double by 2050. Therefore, the prescription of these medications and, consequently, the increase in their side effects is inevitable [3]. A literature review conducted by Kawahara et al. indicated that the incidence of MRONJ ranged from 1% to 2.3% with three years of oral or intravenous bisphosphonate use, while it ranged from 1.3% to 3.2% with three years of anti-RANKL antibody use [4]. Another study revealed that doctors do not have sufficient knowledge about MRONJ and do not address this issue adequately [5].

Apart from the effect of local anatomical structures and relationships in the development of MRONJ, other factors have also been suggested in the literature. Studies have indicated that bisphosphonates may exert a toxic effect on epithelial tissue, and that prolonged use of these drugs can lead to excessive accumulation in the jawbones, enhancing the toxic effect on the oral epithelium. This approach highlights local toxicity, in addition to systemic factors, as a significant contributor in the development of MRONJ [6]. It has been suggested that bisphosphonates accumulated in the bone may lead to toxicity in the overlying mucosa during infection, trauma, surgical treatment, or spontaneous release. This toxic effect can result in the disruption of the soft tissue integrity and the subsequent exposure of the bone. In an in vitro study conducted by Reid, zoledronic acid was found to have a toxic effect by preventing epithelial cells from adhering to bone surfaces. These findings provide significant evidence suggesting that bisphosphonates may have adverse effects on the oral epithelium and contribute to the development of MRONJ [6].

Currently, bisphosphonates are extensively utilized across the globe for the treatment of various conditions, with a particular emphasis on managing osteoporosis [7]. In their studies examining patients between 2003 and 2021, Wadhwa et al. reported that medication was prescribed to 20.6% of patients with osteoporosis [8]. The aim of this study is to evaluate the awareness of specialists who prescribe bisphosphonate drugs regarding MRONJ and their approaches to this condition.

## Material and Method

The study was conducted from February 2024 to April 2024 and the ethics of the study were approved by the Ankara University Health Sciences Sub-Ethics Committee (decision date. 29/01/2024, decision number. 02/05). A total of 172 questionnaires were distributed to physicians working in the Departments of Physical Therapy and Rehabilitation, Oncology, Obstetrics and Gynecology, and Orthopedics and Traumatology at public hospitals, private hospitals, and medical faculties in Ankara. Seven questionnaires were deemed invalid due to missing responses or the selection of multiple options. Additionally, 40 individuals declined to participate in the survey. 125 surveys were deemed valid and included in the study. The purpose and content of the study were explained to the specialist in these departments. Physicians who agreed to participate were asked to sign an informed consent form and complete the questionnaire related to MRONJ. Participants were provided with the questionnaires in person.

**Fig. 1:**
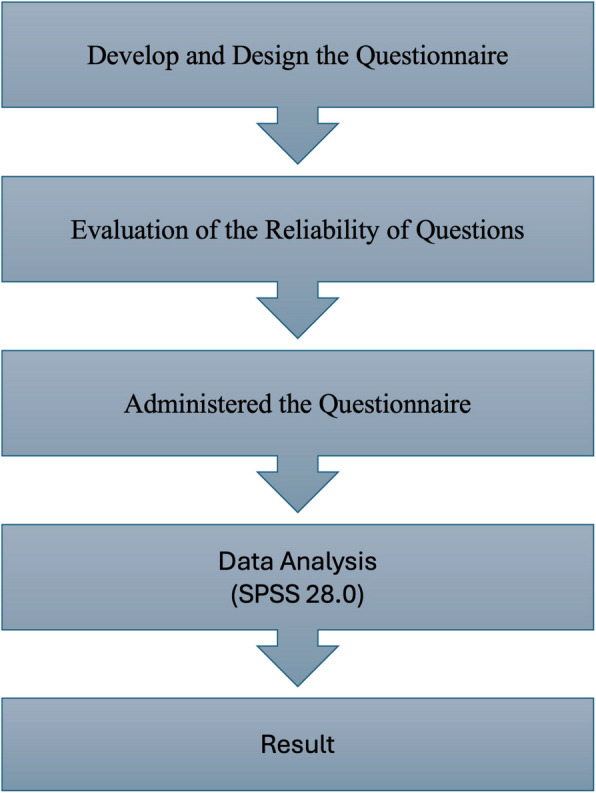
Scheme of the study design

The physicians included in the study were asked to complete a questionnaire containing information such as their specialty, the duration of their practice as specialists, the institution they work for, the number of prescriptions containing bisphosphonates they issue monthly, the most frequently preferred bisphosphonate group and administration method in these prescriptions, the number of consultations they request and receive on this subject monthly, the circumstances under which they seek consultations, whether they consult a dentist for patients who are about to start bisphosphonate therapy, the number of patients with MRONJ they encounter monthly, and the specialty to which they most frequently refer these patients. Additionally, the questionnaire included questions regarding whether physicians request any laboratory values (for instance, Serum CTX) to monitor the adverse effects of bisphosphonates on bone in patients using these drugs, and their approaches when they encounter complications. In this study, a literature review was conducted to prepare the questionnaire, and a new questionnaire was developed based on the one used by Taguchi et al.[9]. Each question prepared in the survey was evaluated by 10 Oral & Maxillofacial Surgeon, and the Content Validity Ratio (CVR) for each question was calculated. Subsequently, the average of the calculated Content Validity Indices (CVI) was taken, resulting in a CVI of 0.98. All questions were calculated using α = 0.05 significance level, and were deemed appropriate based on data specified in the literature [10]. The questionnaire used in our study has been uploaded as Supplementary File 1. The completed questionnaire forms were analyzed and evaluated comprehensively. The stages of the study are shown in Fig. 1.

### Analysis of data

The data obtained from the study were analyzed using the SPSS 28.0 (Statistical Package for Social Sciences) software program. In the study, descriptive statistics (frequency, percentage) of the data were provided. The Chi-square test was used to assess whether there was a statistically significant relationship between the groups and the variables in the study. According to the assumption of the Chi-square test, if more than 20% of the cells have an expected value of less than 5, the Fisher's Exact Chi-square test was used. The probability of a Type I error was set at α = 0.05 for all analyses.

## Result

### Participant Information

**Fig. 2:**
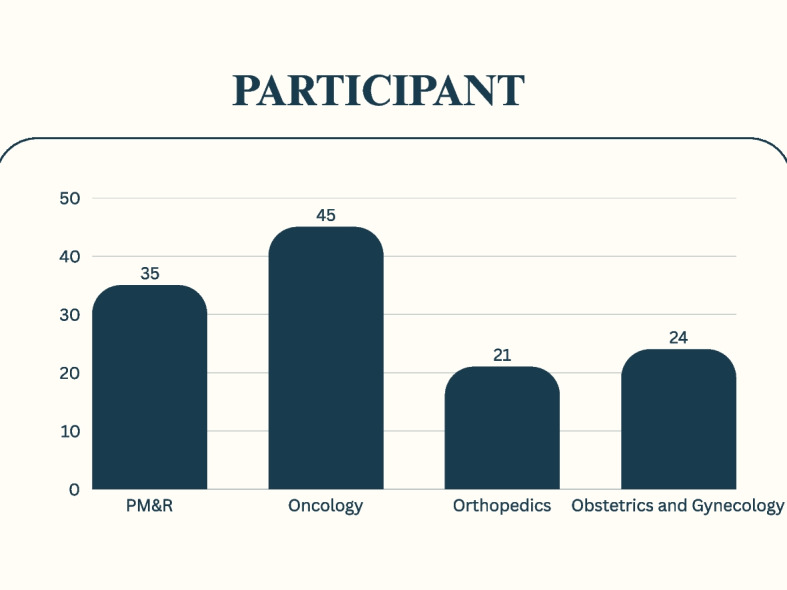
Specialties of the participants

Each response obtained from the surveys was individually evaluated and subjected to statistical analysis. A total of 125 specialist participated in our study, comprising 64 men and 61 women. Among the participants, there are 45 specialists in internal medicine (oncology), 35 specialists in physical medicine and rehabilitation, 24 specialists in obstetrics and gynecology, and 21 specialists in orthopedics and traumatology (Fig. 2). Among the participants in our study, 37.6% (n:47) were specialty residents, while 62.4% (n:78) were specialist doctors. The highest number of specialty residents were in the physical medicine and rehabilitation (PM&R) department, whereas the lowest number of specialist doctors were also in the same department. In terms of professional experience, the shortest duration identified was two-five years, while the longest duration was 21 years. 46.4% (n:58) of the participants work in state hospitals, 31.2% (n:39) in university hospitals, and 22.4% (n:28) in private healthcare institutions. There was no statistically significant difference among participants in terms of age, gender, area of expertise, and the institutions where they work. Additionally, there was no significant difference among doctors regarding seniority or extensive experience in developing sufficient knowledge and cooperation on this subject.

## Participants'Prescription Habits

The monthly prescription rates of bisphosphonate group drugs among the participants were determined as follows: 42.4% (n:53) prescribed in the range of 0–2, 30.4% (n:38) in the range of 3–6, 16.8% (n:21) in the range of 7–12, 5.6% (n:7) in the range of 13–20, and 4.8% (n:6) prescribed more than 20.(Table 1).

**Table 1:** Specialty and the number of antiresorptive prescriptions written monthly

**Antiresorptive prescriptions written monthly**	**Specialty**					**Total**	**X**^**2**^	**p**
	**PM&R**	**Oncology**	**Orthopedics**	**Obstetrics and Gynecology**				
0–2	9	30	8	6		53	48.081	0.000*
	25.7%	66.7%	38.1%	25.0%		42.4%		
3–6	11	13	8	6		38		
	31.4%	28.9%	38.1%	25.0%		30.4%		
7–12	13	1	2	5		21		
	37.1%	2.2%	9.5%	20.8%		16.8%		
13–20	2	0	3	2		7		
	5.7%	0.0%	14.3%	8.3%		5.6%		
More than 20	0	1	0	5		6		
	0.0%	2.2%	0.0%	20.8%		4.8%		
Total	35	45	21	24		125		
	100.0%	100.0%	100.0%	100.0%		100.0%		

*X*^*2*^: Chi-square test statistic, *p < *0.05*

The most commonly preferred administration method was identified as intravenous (IV), by oncology and gynecology specialist.

Alendronat (%50.4 n:63), zoledronat (%40 n:50) and ibandronat (%9.6 n:12) were the most preferable drugs among the physicians.

## Participants'Attitude

In our study, the number of monthly consultations required from different specialists regarding bisphosphonate therapy was as follows: 1–4 consultations at a rate of 60% (n = 75), 0 consultations at a rate of 25.6% (n = 32), 5–10 consultations at a rate of 13.6% (n = 17), and more than 20 consultations at a rate of 0.8% (n = 1).

59.2% (n:74) of the physicians reported that they consult a dentist before initiating bisphosphonate therapy if the patient has a dental issue, while 24% (n:30) of participants stated that they do not deem a dental consultation necessary, 12% (n:15) reported that they request a consultation for every patient to be treated with intravenous (IV) bisphosphonate, and 2.4% (n:3) indicated that they request a consultation for every patient to be treated with oral bisphosphonate prior to the therapy.(Table 2) In the study, most of respondents in each department indicated that they consult a dentist when their patients have a dental problem(59.2%). Orthopedic specialists were the most common consultants to dentists (38.1%) and oncology specialist were the least common consultants (4.4%) prior to the initiation of oral and intravenous bisphosphonate therapy.

**Table 2:** Specialty and the need for consultation with a dentist

In which situations do you need a consultation with a dentist	Specialty				Total	X^2^	p
	**PM&R**	**Oncology**	**Orthopedics**	**Obstetrics and Gynecology**			
I don't need it	4	16	4	6	30	24.364	0.003*
	11.4%	35.6%	19.0%	25.0%	24.0%		
I consult with every patient who will start oral bisphosphonate	0	0	2	1	3		
	0.0%	0.0%	9.5%	4.2%	2.4%		
I consult with every patient who will start IV bisphosphonate	3	2	6	4	15		
	8.6%	4.4%	28.6%	16.7%	12.0%		
If the patient I am starting on bisphosphonates has a dental problem, I will consult	28	26	9	11	74		
	80.0%	57.8%	42.9%	45.8%	59.2%		
Other	0	1	0	2	3		
	0.0%	2.2%	0.0%	8.3%	2.4%		
Total	35	45	21	24	125		
	100.0%	100.0%	100.0%	100.0%	100.0%		

*X*^*2*^: Chi-square test statistic, *p < *0.05*

The number of consultations requested by dentists from physicians per month due to bisphosphonate use was found to be in the range of 1–4 with a rate of 68% (n:85). The lowest number of consultations requested was found to be more than 20 with a rate of 1.6% (n:2). The consultation rate was mostly in the range of 1–4 per month in all specialty; being 76.2% (n:16) in orthopedics, 71.1% (n:32) oncology, 62.9% (n:22) PM&R and 62.5% (n:15) in obstetrics and gynecology.(Table 3).

**Table 3:** Number of monthly consultations requested from different specialties regarding the use of antiresorptive agents

Number of monthly consultations regarding the use of antiresorptive medication	Specialty				Total	X^2^	p
	**PM&R**	**Oncology**	**Orthopedics**	**Obstetrics and Gynecology**			
0	0	13	2_,_	5	20	32.047	0.000*
	0.0%	28.9%	9.5%	20.8%	16.0%		
1–4	22	32	16	15	85		
	62.9%	71.1%	76.2%	62.5%	68.0%		
5–10	6	0	2	2	10		
	17.1%	0.0%	9.5%	8.3%	8.0%		
11–20	6	0	1_,_	1_,_	8		
	17.1%	0.0%	4.8%	4.2%	6.4%		
More than 20	1	0	0	1	2		
	2.9%	0.0%	0.0%	4.2%	1.6%		
Total	35	45	21	24	125		
	100.0%	100.0%	100.0%	100.0%	100.0%		

*X*^*2*^: Chi-square test statistic, *p < *0.05*

The rate of encountering a patient with MRONJ in the past year was found to be zero and between one-four cases for 56% (n:70) and 44% (n:55) of physicians respectively.

The groups that diagnosed MRONJ and requested the most consultations from physicians were determined to be dentists, accounting for 50.4% (n:63), and oral and maxillofacial surgeons, accounting for 36.8% (n:46). (Table 4).

**Table 4:** Specialty requiring consultation for patients with MRONJ

Specialty who dianosed MRONJ and refferred for consultation	Referred Specialty				Total	X^2^	p
	**PM&R**	**Oncology**	**Orthopedics**	**Obstetrics and Gynecology**			
Dentistry	11	27	13	12	63	10.089	0.280
	31.4%	60.0%	61.9%	50.0%	50.4%		
Ear-Nose-Throat	4	3	2	3	12		
	11.4%	6.7%	9.5%	12.5%	9.6%		
Plastic and Reconstructive Surgery	2	1	1	0	4		
	5.7%	2.2%	4.8%	0.0%	3.2%		
Oral and Maxillofacial Surgery	18	14	5	9	46		
	51.4%	31.1%	23.8%	37.5%	36.8%		
Total	35	45	21	24	125		
	100.0%	100.0%	100.0%	100.0%	100.0%		

*X*^*2*^*: Chi-square test statistic*

In our study, the percentage of specialists who utilized biochemical tests to assess the risk of osteonecrosis was 36% (n = 45). The proportion of those who responded"sometimes"was 26.4% (n:33), and those who answered"no"constituted 37.6% (n:47).

39.2% (n:49) of physicians reported referring a patient with MRONJ to an oral and maxillofacial surgeon. Meanwhile, 19.2% (n:24) indicated that they take a break from the medication, 15.2% (n:19) referred the patient to an ENT specialist, 6.4% (n:8) to plastic surgery specialist and other 17.6% (n:22) stated that they stopped medication. (Table 5).

**Table 5:** Specialty and approach of specialist to MRONJ

Approach of specialist to MRONJ	Specialty				Total	X^2^	p
	**PM&R**	**Oncology**	**Orthopedics**	**Obstetrics and Gynecology**			
I’ll stop medication	16	6	0	0	22	39.639	0.001*
	45.7%	13.3%	0.0%	0.0%	17.6%		
I'll take a break from the medication	9	7	5	3	24		
	25.7%	15.6%	23.8%	12.5%	19.2%		
I refer the patient to a maxillofacial surgeon	6	20	11	12	49		
	17.1%	44.4%	52.4%	50.0%	39.2%		
I refer the patient to an ENT	2	9	3	5	19		
	5.7%	20.0%	14.3%	20.8%	15.2%		
I refer the patient to a Plastic Surgery	1	3	1	3	8		
	2.9%	6.7%	4.8%	12.5%	6.4%		
Other	1	0	1	1	3		
	2.9%	0.0%	4.8%	4.2%	2.4%		
Total	35	45	21	24	125		
	100.0%	100.0%	100.0%	100.0%	100.0%		

*X*^*2*^: Chi-square test statistic, *p < *0.05*

## Discussion

Despite the recent advancements in the pharmacological treatment of MRONJ, an effective treatment plan is still lacking for the troubling side effects of the drugs that lead to this condition. Due to the distinctive effects and accumulation of these drugs in the jawbones, differing from more than other bones, severe complications may arise, particularly following oral surgical procedures. To prevent this condition, it is critically important to enhance the awareness of both dentists and the physicians prescribing these medications, and develop a collaborative treatment plan.

The most commonly used bisphosphonate drug was found to be alendronate in this study. The highest incidence of MRONJ being associated with zoledronate and alendronate can also be attributed to these two drugs being the most frequently used bisphosphonates. According to the results of a literature analysis study involving 371 patients diagnosed with MRONJ, 68% were using zoledronic acid, 14% were using alendronate, and 18% were using other antiresorptive medications [11]. In the study conducted by Sentürk et al., 98.1% of the participating oncologists preferred to use zoledronate [12]. In another study, medical oncologists were found to prefer zoledronic acid for intravenous administration and ibandronate for oral administration [13].

In our study, the most commonly preferred administration method was intravenous by oncology and gynecology physicians. The preferrence for IV administration and zoledronate can be explain by the fact that this method provides higher bioavability and results more rapidly and effectively. PM&R specialist were the group who most frequently preferred oral administration. Oral administration for the treatment of mild or moderate osteoporosis should be suggested to reduce the serious side effect of bisphosphonates in jaw bones.

Similar to our study, Karen et al. found that 71.2% of patients who developed MRONJ received antiresorptive drugs intravenously (IV), while 28.8% received them orally. In another study, 40% of MRONJ patients were found to be using oral bisphosphonates for osteoporosis treatment, while 60% were receiving the drug intravenously (IV) due to various malignancies [14, 15].

In a study conducted by Kim et al. on physicians'perceptions of MRONJ, 57.8% of participants prescribed bisphosphonate drugs to 1–11 patients per month, 13.5% to 11–20 patients, and 28.7% to more than 21 patients [14]. In another study on physicians'awareness of MRONJ, 74.4% of the participants reported prescribing bisphosphonate drugs to 1–5 patients per month, while 16.2% prescribed them to 6–10 patients, and 9.4% prescribed them to more than 11 patients [16]. In the present study, the number of patients prescribed bisphosphonates in a month was found to be in the range of 0–2 with 42.4% rate.

In 2005, the FDA provided information on the complications of bisphosphonates to increase awareness of MRONJ among healthcare professionals. However, in a study conducted by Kim et al. in 2016 on MRONJ awareness, 21.9% of participants stated that they had never heard of this condition before, and only 9.9% were able to answer the questions correctly. Despite the relatively low awareness of MRONJ, the monthly prescription rates of antiresorptive drugs by physicians were reported as follows: 57.8% 1–11 prescriptions, 13.5% 11–20 prescriptions, and 28.7% more than 21 prescriptions. Additionally, the awareness of MRONJ was found to be highest among oncologists and lowest among orthopedic surgeons [14]. In a survey conducted by Osta et al. with 190 participants, the awareness of MRONJ among medical doctors was evaluated, and 38% of participants had no knowledge of MRONJ [17]. As seen from the results of these studies, there is a need to increase the awareness and selectivity of doctors who prescribe antiresorptive drugs.

According to the study conducted by Şenol et al., 21.4% of physicians always recommended dental treatment to patients before starting antiresorptive drugs, 51.4% referred patients to a dentist only if there was a dental complaint, and 27.3% did not refer their patients to a dentist at all. In the same study, the majority of physicians (62.6%) indicated that they referred their patients to a dentist for dental examination only in the presence of dental complaints [18]. In a survey conducted by Şentürk et al. involving 60 oncologists, only 62% of the specialists referred their patients for dental examinations prior to initiating IV bisphosphonate treatment, while 36% believed that such consultations were unnecessary [12]. In our study, most of the participants (59.2%) indicated that they refer patients to a dentist before starting this type of medical treatment only if there is a dental problem. 24% of our participants especially the medical onchologists mentioned that they didn’t need dental consultation. This may be attributed to underestimating oral complications when faced with life-threatening health problems of a malingnancy. In this regard, it is crucial for physicians to provide informative presentations and publications about the potential issues in the jaws and oral tissues associated with antiresorptive drugs, as well as the preventive measures that should be taken.

A recent clinical practice statement issued by the American Academy of Oral Medicine highlighted the lack of sufficient evidence to support the use of CTX as a predictive biomarker for assessing the risk of MRONJ, especially in patients receiving intravenous bisphosphonates or denosumab following dental procedures [19]. According to a study conducted by Şenol et al., the use of biochemical tests (e.g., CTX) to assess the risk of osteonecrosis in patients receiving antiresorptive therapy was investigated. 55% of the participants stated that they do not resort to these tests, 36.4% said they sometimes do, and 8.2% said they always do [18]. Serum CTX levels alone are not a reliable predictor or preventive measure to prevent complications. Other biomarkers have been suggested for the prediction of MRONJ. The serum levels of bone alkaline phosphatase (BAP), type I collagen N-terminal telopeptide (NTX), parathyroid hormone (PTH), deoxypyridinoline (DPD), osteocalcin (OCN), and pyridinoline (PYD) have been investigated [20]. In present survey, no significant difference was found among physicians from different specialties regarding the rate of ordering biochemical tests. It can be inferred that there is no common consensus among physicians on this subject. It is noteworthy that the proportion of physicians who resort to biochemical tests is low. Although it is not a definitive marker, dentists should resort to this test and appreciate its importance, especially in dental implant planning or minor surgeries involving the bone.

The specialists who participated in the study were more likely to refer patients to oral and maxillofacial surgeons (39.7%) when they observe MRONJ. In a study, the treatment options preferred by oncology specialists in cases of MRONJ were examined. According to the study, 11.3% of the specialists reported that they immediately discontinued the drug, 52.8% paused the drug, 7.5% adjusted the dosage, 15% paused the drug in the event that lesions persisted despite dosage adjustment, and 3.7% reported changing the drug [12]. A drug holiday is considered one of the foremost methods thought to be effective in the treatment of cases where MRONJ has developed. In a study conducted by Dickinson et al., the vast majority of specialists recommended a temporary discontinuation of the drug for the surgical treatment of MRONJ in their cases. However, short-term drug holidays have been inadequate in providing noticeable improvement. While longer-term drug holidays have been observed to stabilize the necrotic area, this situation may increase the patient's risk for skeletal complications and metastasis [21].

Although our study reflects the attitude of physicians when they encounter MRONJ to some extent, it should not be ignored that there are some limitations. In order to raise awareness about MRONJ and reduce its prevalence, conducting studies with more participants and addressing more diverse issues will help to fill the gaps in knowledge on this subject.

As oral surgical procedures such as dental extractions, mucosal irritations and dehiscencies are the predisposal factors of MRONJ, oral and maxillofacial surgeons takes an important role in the management of such cases. Therefore specialist administrating bisphosphantes and other antiresorptive or antiangiogenetic drugs should always work collaboratively with oral and maxillofacial surgeons and dentists for prevention and treatment of these cases.

## Conclusion

In patients using bisphosphonates and antiresorptive drugs, the importance of collaboration between physicians and dentists, as well as pre-treatment dental consultation and cooperation, is unfortunately often overlooked during the diagnosis and treatment process of the primary disease. Most patients who use bisphosphonates during the treatment of their primary disease encounter dental issues and consult a dentist. Typically, these patients who have inadequate oral hygiene, face the necessity for extensive periodontal treatment or the requirement for one or more tooth extractions. Such interventions in the oral region can lead to an increased risk of osteonecrosis. To enhance awareness of MRONJ, a multidisciplinary approach should be adopted between the medical and dental disciplines, and a well-coordinated consultation system should be established. Before initiating antiresorptive drug therapy, it is ensured that patients undergo dental examinations and complete any necessary interventional dental procedures. In addition, the implementation of educational strategies such as academic publications, educational seminars, and symposiums will be beneficial in raising awareness and addressing gaps in this area.

## Clinical trial number

Not applicable.

## Supplementary Information

Supplementary Material 1.

## Data Availability

The data supporting this study’s findings are available from the corresponding author upon reasonable request.

## Abbreviations

MRONJ: Medication-Related Osteonecrosis of the Jaw
BRONJ: Bisphosphonate-related osteonecrosis of the jaw
AAOMS: American Association of Oral and Maxillofacial Surgeons
CTX: C-terminal telopeptide
CVR: Content Validity Ratio
CVI: Content Validity Indices
IV: Intravenous
P: Probability value
X^2^: Chi-squared test
