# Supplementary material for: Awareness and Attitude Towards MRONJ Among Physicians Prescribing Antiresorptive Drugs: A Cross-Sectional Study
Source: BMC Oral Health. 2025 Jul 2;25:985. doi: 10.1186/s12903-025-06490-5 (PMC12217535; doi:10.1186/s12903-025-06490-5)
Supplement: Supplementary file 1 — Supplementary Material 1. [file 12903_2025_6490_MOESM1_ESM.pdf]

Evaluation of Medical Doctors’ Approaches to Medication-Related Osteonecrosis of the Jaw

1. Gender:

    a) Female b) Male

2. Specialization:

    a) Physical medicine and rehabilitation
    b) Oncology
    c) Orthopedics
    d) Obstetrics and Gynecology

3. Position:

    a) Resident b) Specialist

4. How long have you been working in your profession?

    a) 0-2 years b) 2-5 years c) 5-10 years d) More than 10 years

5. What institution do you work for?

    a) University Hospital b) State Hospital c) Private Hospital d) Medical Practice

6. What is the number of bisphosphonate prescriptions you write monthly?

    a) 0-2 b) 3-6 c) 7-12 d) 13-20 e) More than 20

7. What is the most commonly prescribed form of bisphosphonate?

    a) 100% IV – 0% Oral
    b) 80% IV – 20% Oral
    c) 60% IV- 40% Oral
    d) 40% IV- 60% Oral
    e) 20% IV- 80% Oral
    f) 0% IV – 100% Oral

8. What is the bisphosphonate group of medication you use most often?

a) Etidronate
b) Tiludronate
c) Aledronate
d) Risudronate
e) Ibandronate
f) Pamidronate
g) Zoledronate

9. What is the number of consultations you perform monthly due to the use of bisphosphonates?

a) 0 b) 1-4 c) 5-10 d) 11-20 e) More than 20

10. In which cases do you need a dental consultation?

a) I do not need
b) In every patient in whom I will start oral bisphosphonates
c) every patient in whom I will start intravenous bisphosphonates
d) f there is a dental problem in the patient in whom I will start bisphosphonates
e) Other

11. What is the number of monthly consultations you receive regarding the use of bisphosphonates?

a) 0 b) 1-4 c) 5-10 d) 11-20 e) More than 20

12. What is the number of patients you encountered in a year with medication-related osteonecrosis?

a) 0 b) 1-4 c) 5-10 d) 11-20 e) More than 20

13. Which specialties most commonly refer patients with medication-related osteonecrosis to you?

a) Dentists
b) Ear- Nose- Throat
c) Plastic and Reconstructive Surgery
d) Infectious Diseases
e) Oral, Dental and Maxillofacial Surgery

14. Do you use biochemical tests (such as CTX, etc.) to assess the risk of developing osteonecrosis?

a) Yes   b) No   c) Sometimes

15. What is your approach when you encounter a patient with medication-related osteonecrosis?

a) stop the medication.
b) I take a break from medication.
c) I refer the patient to oral and maxillofacial surgery
d) I refer the patient to ENT (Ear, Nose, Throat) specialist
e) I refer the patient to plastic surgery.
f) I treat the patient with my own treatment method
g) Other
